# From endemic shadows to the light of dawn: the 120-year journey of China's anti-schistosomiasis chariot

**DOI:** 10.1186/s40249-025-01395-5

**Published:** 2025-11-30

**Authors:** Shan Lv, Li-Gang Zhou, Jing Xu, Shi-Zhu Li, Robert Bergquist, Jürg Utzinger, Xiao-Nong Zhou

**Affiliations:** 1 National Institute of Parasitic Diseases, Chinese Center for Disease Control and Prevention (Chinese Center for Tropical Diseases Research); Key Laboratory On Parasite and Vector Biology, National Health Commission; WHO Collaborating Centre for Tropical Diseases; National Center for International Research on Tropical Diseases, Ministry of Science and Technology, Shanghai, PR China; 2 Geospatial Health, Brastad, Sweden; 3 Swiss Tropical and Public Health Institute, Allschwil, Switzerland; 4 University of Basel, Basel, Switzerland; 5 School of Global Health, Chinese Center for Tropical Diseases Research, Shanghai Jiao Tong University School of Medicine, Shanghai, PR China

## Abstract

Schistosomiasis was first reported in the People's Republic of China (P.R. China) 120 years ago. The national programme of schistosomiasis control started in mid-1950s. The transmission interruption was achieved by 2023. Here we summarize the experience as the five core parts of a running chariot. While the specific historical context of P.R. China's way of schistosomiasis control may be difficult to replicate, the experiences gained underscore several critical principles that might be applicable elsewhere.

**Graphical Abstract:**

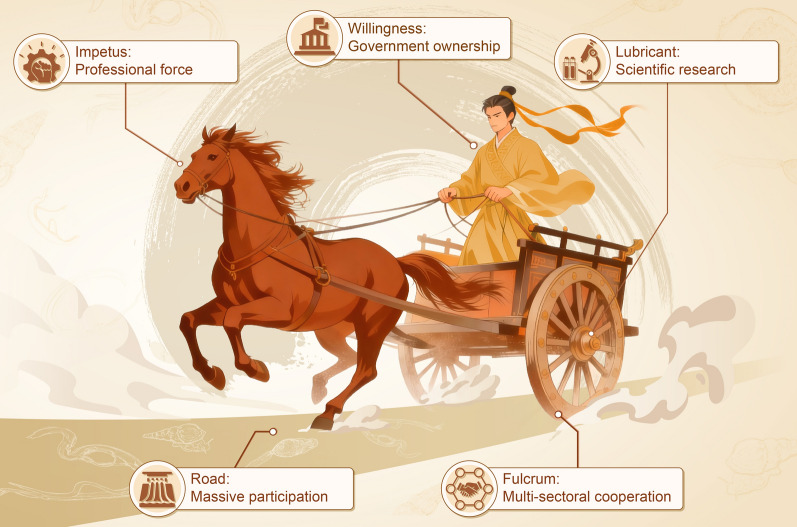

## Brief history

One hundred and twenty years ago, the first report of human schistosomiasis in the People's Republic of China (P.R. China) was published in the *China Medical Missionary Journal* (in 1907 renamed the *Chinese Medical Journal*) by Dr. O. T. Logan, an American physician working in China [1]. Henceforth, the disease was reported in several provinces and in 1924, Faust and Meleney were the first to provide details of its distribution in the country [2]. Towards the end of the 1920s, the Chinese central government started to conduct surveys and mounted efforts for the control of schistosomiasis [3]. The initial surveys revealed that the disease was endemic in the Yangtze River Delta, with an estimated 5 million infections, while tens of millions of cases were projected for the entire country [4]. Despite these estimates and the considerable burden caused by schistosomiasis, no national programme was implemented during the war years.

Importantly, schistosomiasis was recognized as a major public health problem just before the founding of P.R. China in 1949. To wit, more than 30,000 soldiers of the People's Liberation Army were weakened by *Schistosoma* infection [5]. Particularly high endemicity was reported along the Yangtze River later. In the early 1950s, specialized agencies for schistosomiasis control were established, with morbidity control as the key objective. The Leading Group for Schistosomiasis Control of the Central Committee of the Communist Party of China (CPC) was established in 1955, which marked the launch of the national schistosomiasis control programme [6]. Within 3 years, the first nationwide survey was carried out and reported over 9 million cases in 12 provincial-level administrative divisions (PLADs) [7]. Meanwhile, endemic counties took various measures to treat infected people and eliminate the intermediate host snails. In 1958, Yujiang County, located in Jiangxi Province, declared the elimination of schistosomiasis [8]. When this important news was presented to Chairman Mao Zedong, who was also known as a great poet, he was overcome by joy and completed the now famous poem ‘Farewell to the God of Plague’ overnight [9]. Today, this poem still encourages public health specialists and endemic communities to fight against schistosomiasis in P.R. China.

From the early start of the schistosomiasis control programme, it was integrated into the national agriculture development schemes [10]. After all, those most affected by schistosomiasis were the farmers and their domestic animals. In addition, irrigation development and water conservancy facilitated the fight against *Oncomelania hupensis*, the only intermediate host snail species of *Schistosoma japonicum*. *O. hupensis* was first discovered in Hubei province in 1881. The amphibious features make *O. hupensis* is sensitive to the soil humidity [11]. Any environmental changes that affect soil humidity can destroy habitats. The first national 5-year strategic plan for schistosomiasis control was presented in 1966 [12]. Given the success of this 5-year strategic plan, these plans have continuously been renewed to date.

Owing to the massive campaigns against schistosomiasis and the large-scale application of the antischistosomal drug praziquantel, the prevalence of schistosomiasis declined to very low levels already in the early 1980s. By the mid-1980s, four out of 12 endemic PLADs (i.e. Guangdong, Shanghai, Fujian and Guangxi) had declared the elimination of schistosomiasis [13]. The leadership in schistosomiasis control shifted from CPC to the State Council (more precisely the Ministry of Health), which took over the responsibility for schistosomiasis control in 1986. Flooding events in the endemic area adversely affected schistosomiasis control. Two far-reaching flooding events in 1989 and 1998 drew attention of the State Council as they caused the prevalence to rebound. In response, various multi-sectoral cooperation schemes were established, while funding shortages were addressed through external funding. By joint funding at CNY 890 million (around 150 million USD at the time) by the central government and the World Bank, a 10-year project to fight schistosomiasis was set up [14]. This World Bank Loan Project (WBLP), still the largest and most costly single control project ever funded, commenced in 1992 and emphasized mass drug administration (MDA) with praziquantel coupled with information, education and communication (IEC) campaigns. Since 2004, a new integrated strategy focusing on the control of infection sources was applied nationwide and lead to nationwide transmission interruption by 2023, which means no newly infection was found in humans, domestic animals or snails in the last five consecutive years [15]. The target of elimination will be achieved when the transmission interruption maintained for at least another five consecutive years. All of 27 thousand advanced human cases, who do not release alive eggs of *Schistosoma* any more, are supplied with free treatment. Despite the overall great success achieved, the vast distribution of *Oncomelania* snails and various mammalian reservoirs (water buffaloes in particular) are challenging and the last mile of schistosomiasis elimination is still ahead of us.

## Strategy evolution

**Fig. 1:**
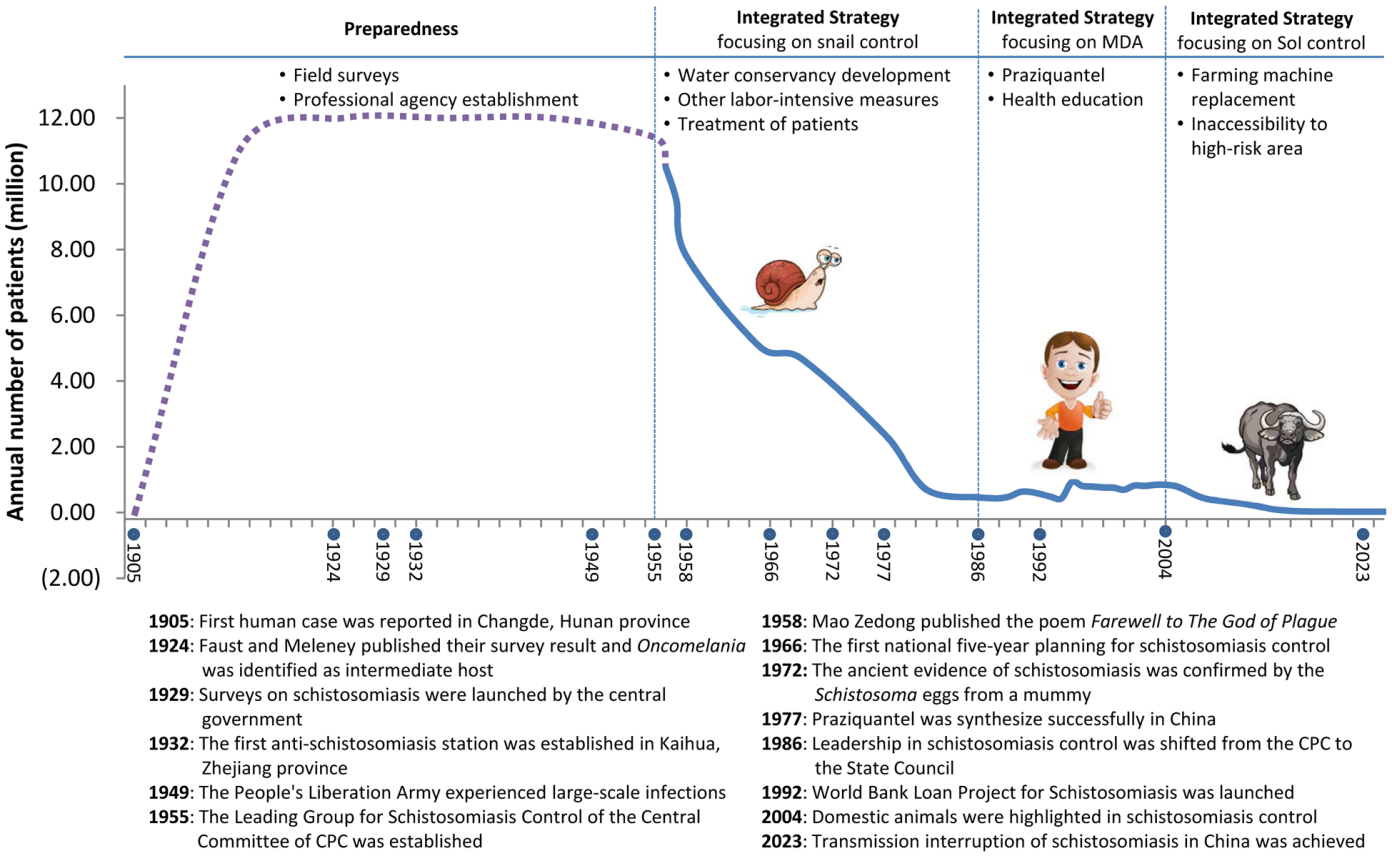
The prevalence of human schistosomiasis in P.R. China in the past 120 years with major events

Four stages can be identified in the 120-year history from the first written report of schistosomiasis towards disease elimination in P.R. China (Fig. 1). The first stage between 1905 and 1954 was the period of preparedness. The public health importance of schistosomiasis was recognized gradually. Professional agencies, including research institutes and designated schistosomiasis hospitals at various administration levels, were established. These developments went hand-in-hand with capacity strengthening. Morbidity control was the declared objective. In parallel, snail control methods were developed and validated. The second stage between 1955 and 1985 was based on an integrated strategy focusing on snail control. The rural collective economy was developed in this period. Labour-intensive reclamation of farmland, development of irrigation systems and water management led to a significant decline in the distribution range of *O.* *hupensis* [16]. As a consequence, the prevalence in humans decreased considerably [17]. The third stage began in 1986 and lasted until 2003. The reform of the rural economic system was completed in the early 1980s. The leadership of schistosomiasis control was shifted from the CPC to the Central Government (i.e. the State Council) in 1986. Praziquantel with its proven safety and efficacy profile was widely administered and further intensified by the WBLP (1992–2002) [14]. Indeed, MDA with praziquantel was the core of strategy of the schistosomiasis control strategy at this stage.

Schistosomiasis control entered the fourth stage starting in 2004, which brought vast changes. This was due to the realization that further lowering of human schistosomiasis prevalence had encountered problems, with flooding events extending the range of *O. hupensis* and schistosomiasis in the domestic animals imposing the risk of re-transmission involving humans [18]. Controlling the sources of infection, particularly infected cattle, became the crucial measure used to fight against schistosomiasis [19]. The use of water buffaloes for ploughing was gradually replaced by mechanized agriculture, and roaming cattle were forbidden to enter grasslands infested by *O. hupensis*. All these activities were guaranteed by new regulations and social and economic investment.

## Anti-schistosomiasis chariot

**Fig. 2:**
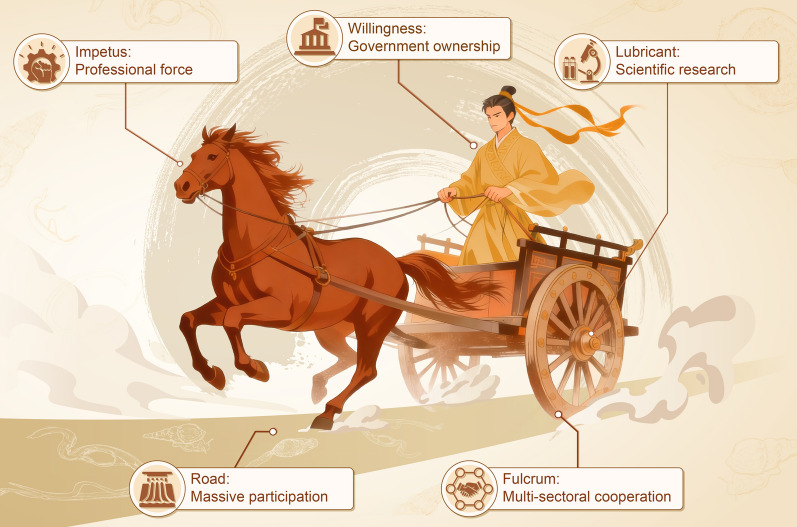
A running chariot denoting the five core components of anti-schistosomiasis campaign in P.R. China

Although the first case of schistosomiasis japonica was reported 120 years ago, it took half a century to mount the national schistosomiasis control programme. In the remainder of this paper, we summarize the experience of schistosomiasis control in P.R. China over the past 70 years emphasizing five essential parts without which this chariot could not have moved forward (Fig. 2).

### Government ownership

Of note, schistosomiasis was never a neglected disease in P.R. China. Instead, it was considered one of the top diseases to be eliminated in the first National Agriculture Development Outline (1956–1967). Moreover, it was the only disease in whose management the Central Committee of CPC directly intervened until 1986 when the Leading Group for Schistosomiasis Control of the Central Committee of CPC was dismissed. CPC had successfully initiated social mobilization to fight against schistosomiasis in the era when the economy was still weak. It was strongly involved in the first systematic survey on schistosomiasis and *Oncomelania* snails that was carried out between 1956 and 1958. Schistosomiasis control then became a priority consideration when developing irrigation system and water conservancy projects in endemic areas. The massive campaign against schistosomiasis is described in Mao Zedong’s poem ‘Farewell to the God of Plague’.

Although the leadership shifted from the Central Committee of CPC to the State Council in 1986, the national programme for schistosomiasis control developed gradually owing to continuous investment from both central and local governments’ budget. The only foreign investment into schistosomiasis control came from the World Bank from 1992 to 2002. Although the WBLP emphasized MDA coupled with IEC, the Chinese government never gave up snail control and continuously invested in this and multisectoral interventions, which contributed to keeping the integrated strategy moving forward.

### Professional workforce

The implementation of a national programme was essential for schistosomiasis control. A skilled workforce was the pre-condition for such a programme. The system of schistosomiasis control, including leading groups, special agencies, research committees and multisectoral cooperation, from central to local governments formed in the late 1950s shortly after the foundation of the leading group of the Central Committee of CPC in 1955. By the end of 1957, there were 17 institutions, 180 stations, 1282 teams and 16,722 professionals dedicated to fight human schistosomiasis in China [7]. In the most highly endemic areas, 12 special hospitals for schistosomiasis with 21,610 beds were established [7]. Clinics for schistosomiasis in general hospitals were also common. Many physicians were involved in treatment of schistosomiasis in the early campaign.

Once elimination of schistosomiasis in a given setting was achieved, specialized schistosomiasis institutions and stations were merged into local centres for disease control and prevention. However, the capacity for surveillance is maintained in each of the previously endemic counties. According to a survey in 2023, there were 486 agencies with specific functions to advance schistosomiasis surveillance and control, which included 5,675 professionals [20].

### Multisectoral cooperation

The lifecycle of *S.* *japonicum* is more complex than that of other human *Schistosoma* species, since it is a zoonosis including more than 40 mammalian species acting as reservoir hosts. In addition to humans, domestic animals such as cattle, water buffaloes and pigs were also susceptible to the parasite. In addition, *Oncomelania* snails were affected by farm reclamation and irrigation systems. Need for an integrated strategy for schistosomiasis control was recognized already in the 1930s [21]. Positive experience with an integrated control approach at county level were made in Yujiangin the 1950s [8]. The integrated strategy had four main pillars: patient treatment, snail control, faeces management and health education [22].

The Ministry of Agriculture as well as the Ministry of Health, the Ministry of Education and All-China Women's Federation were the earliest members of the leading group of the Central Committee of CPC. Hence, the multi-sectoral cooperation had been established at the beginning of the national control programme. Later on, additional sectors, including the Ministry of Water Resources, the Ministry of Transport, Ministry of Forestry, the Ministry of Finance and the National Development and Reform Commission, were involved. Similar organizations were set up at the province, city and county levels. Up to date, this multisectoral cooperation is still working.

### Scientific research

No alternative drug has replaced praziquantel since the early 1980s, this drug has been the cornerstone of schistosomiasis control for over 40 years as recommended by the World Health Organization (WHO). Its guidelines for schistosomiasis control further recommend praziquantel as the sole drug for treating schistosomiasis and niclosamide as the only compound for snail control. However, prior to the availability of these safe and efficacious interventions, there were a host of technological innovations that advanced schistosomiasis control in the initial phase of the programme. For example, various Chinese herbs (e.g. *Artemisa annua*, *Agrimonia pilosa*, *Hemerocallis thunbergii*) and compounds (e.g. furapromide, amoscanate, sodium antimony gallate) were tested for their therapeutic efficacy, and some of them were scaled up to treat patients [23]. Novel diagnostic methods (e.g. miracidial hatching, cercarien-Hüllen reaction) were developed and even applied in national surveys [23]. For infection prevention, snail elimination was a crucial strategy. A wide range of snail control methods, including ecological approaches, chemical molluscicides and various plant extracts, were extensively tested and applied from the 1950s to the 1970s. Furthermore, new technologies such as an enzyme-linked immunosorbent assay and remote sensing were rapidly introduced and scaled and continuously refined in response to the changing epidemiology.

Beyond technological advances, continuous innovation in prevention and control strategies were implemented to adapt to varying epidemic conditions across different stages and regions to enhance effectiveness. For instance, following successful trials in controlling infection sources, the approach was scaled up nationwide and formally incorporated into the national plan [24]. Innovations in both technology and strategy typically significantly boost prevention and control efficiency. It should in this connection be added, that a few percent (3.5%) of the total funding available for the WPLP were used for research, and it can be said that the results achieved have in fact prolonged the impact of the WPLP into today’s control programmes [14, 25].

### Massive participation

Social mobilization proved highly effective in P.R. China's schistosomiasis control efforts from the 1950s to the 1970s. During this period, the development of a rural collective economy—characterized by communal ownership and management of assets, with profits distributed among farmers—enabled mass participation. Millions of farmers in endemic areas joined the national campaign, undertaking labour-intensive environmental modifications. These included developing irrigation systems, transforming land, and constructing reservoir dams, actions that collectively altered *Oncomelania* snail habitats and reduced snail density. Simultaneously, farmers served as both recipients of IEC targeting schistosomiasis and agents of sanitation promotion. Their extensive involvement largely addressed the shortage of professionals and was instrumental to the programme’s success.

Following rural economic reforms initiated in 1978 and largely completed by the mid-1980s, large-scale participation in major water conservancy and land reclamation projects diminished in the new economic era. However social mobilization for disease screening and treatment persists. Sustained community cooperation facilitates ongoing annual surveillance, which currently screens approximately 4 million people and over 100,000 cattle and water buffaloes.

## Outlook

While the specific historical context of P.R. China’s way of schistosomiasis control may be difficult to replicate, the experiences gained underscore several critical principles that might be applicable elsewhere, not only schistosomiasis but also other neglected tropical diseases.

First, government ownership cannot be overemphasized. National and sub-national governments must develop, resource, and consistently implement medium- and long-term control plans with robust monitoring and evaluation frameworks. Government long-term will is essential for mobilizing cross-sectoral resources and coordinating a multi-faceted response that is readily adopted to social-ecological settings.

Second, the One Health approach or integrated strategy is fundamental. Schistosomiasis, while a major public health issue, requires interventions beyond the health sector. Endemic countries must establish a high-level, cross-ministerial coordination mechanism with the authority to integrate efforts across health, water, sanitation, agriculture, and education.

Third, a professional workforce is indispensable for effective control. Project-based health workers should be formally integrated into the national health system. Sustained progress towards control or elimination goals ultimately depends on skilled personnel to implement, manage, and adapt the national programme. In addition, professional workforce should play a key role in the innovation and implementation of technologies and strategies.

## Data Availability

Not applicable.

## Abbreviations

P.R. China: the People’s Republic of China
CPC: Communist Party of China
PLADs: Provincial-level administrative divisions
MDA: Mass drug administration
WBLP: World Bank Loan Project
IEC: Information, Education and Communication
